# The Natural Chemotherapeutic Capsaicin Activates AMPK through LKB1 Kinase and TRPV1 Receptors in Prostate Cancer Cells

**DOI:** 10.3390/pharmaceutics14020329

**Published:** 2022-01-29

**Authors:** Belén G. Sánchez, Alicia Bort, José M. Mora-Rodríguez, Inés Díaz-Laviada

**Affiliations:** 1 University of Alcalá, School of Medicine and Health Sciences, Department of Systems Biology, Biochemistry and Molecular Biology Unit, 28871 Alcalá de Henares, Madrid, Spain; belen.sanchezg@edu.uah.es (B.G.S.); alicia.bort@uah.es (A.B.); josem.mora@uah.es (J.M.M.-R.); 2 Chemical Research Institute “Andrés M. del Río” (IQAR), Alcalá University, 28871 Alcalá de Henares, Madrid, Spain

**Keywords:** capsaicin, AMPK, LKB1, TRPV1, PC3, LNCaP, DU-145, prostate cancer

## Abstract

The natural bioactive compound capsaicin has been reported to have anticancer activity, although the underlying mechanism of action has not been completely clarified. Herein, we investigated the mechanism whereby capsaicin exerts antitumor effects on prostate cancer cells. We found that capsaicin activated AMP-activated kinase (AMPK) and promoted cell death in the LKB1-expressing prostate cancer cell lines LNCaP and PC3, but not in the liver kinase B1 (LKB1)-null cell line DU-145. Capsaicin treatment stimulated LKB1 phosphorylation and activated AMPK in LKB1-expressing cells. In addition, LKB1 silencing in LNCaP and PC3 cells abrogated capsaicin-induced AMPK activation, while the overexpression of LKB1 by lentiviral infection in DU-145 cells induced capsaicin-triggered AMPK phosphorylation. Moreover, the calcium/calmodulin-dependent kinase kinase 2 (CaMKK2) inhibitor STO-609 did not modify the activation of AMPK induced by capsaicin, suggesting a CaMKK2-independent mechanism. Capsaicin-induced LKB1 phosphorylation was dependent on the transient receptor potential cation channel subfamily V member 1 (TRPV1), since TRPV1 knocked down by shRNA abolished LKB1 and AMPK phosphorylation in LKB1-expressing cells. Altogether, our results showed that capsaicin affected AMPK activity in an LKB1- and TRPV1-dependent fashion, linking TRPV1 with cell fate. These data also suggest that capsaicin may be a rational chemotherapeutic option for prostate tumors.

## 1. Introduction

In recent years, natural compounds have gained notable attention in the development of new therapeutic interventions in cancer, as they can selectively target numerous signaling pathways implicated in tumor development and progression [1]. In this line, we have shown that the spicy ingredient extracted from peppers belonging to the Solanaceae family, capsaicin, exerts antitumor effects in prostate cancer, acting synergistically with docetaxel to inhibit prostate cancer growth [2,3,4]. Capsaicin has been shown to be more efficacious than other natural compounds at inhibiting prostate cancer cell proliferation [5]. Capsaicin induces autophagy blockage and apoptosis in prostate cancer PC-3 cells [4,6], inhibits the growth of castration-resistant prostate cancer cells [7,8] and causes the degradation of the androgen receptor (AR) [9]. In addition, it has been shown to sensitize human prostate cancer cells to radiotherapy [10] and reduce metastasis in the transgenic adenocarcinoma of the mouse prostate model [11]. Although capsaicin targets the transient receptor potential cation channel subfamily V member 1 (TRPV1) and inhibits the oxidoreductase tNOX [12], the underpinning mechanism involved in its antiproliferative effect in prostate cells remains elusive [13]. We recently determined that the tumor suppressor properties and synergic efficacy of capsaicin were associated with AMP-activated kinase (AMPK) activation [2,14]; however, the mechanism used by capsaicin to activate AMPK remains unknown.

AMPK has been revealed as a relevant target in cancer with both beneficial and adverse roles [15]. Although it has been extensively demonstrated that AMPK activation protects from cancer incidence and behaves as a tumor suppressor, in some cases it can sustain cell growth and protect against the metabolic stress that cancer cells undergo [15]. Evidence supporting the beneficial role of AMPK activation in prostate cancer comes from patients with type 2 diabetes mellitus who were treated with metformin, an activator of AMPK [16,17,18,19,20]. Patients treated with metformin had a lower prostate cancer incidence [16,17] and a better response in survival and recurrence [18]. A negative association between serum prostate-specific antigen (PSA) levels and metformin use has also been observed in prostate cancer patients [19]. In prostate cells, metformin suppresses androgen receptor activation and signaling pathways involved in cell growth and proliferation [20]. 5-aminoimidazole-4-carboxamide riboside (AICAR), another well-known activator of AMPK, induces apoptosis, inhibits the migration and invasion of prostate cancer cells [21] and sensitizes cells to radiotherapy [22]. Likewise, nummularic acid, extracted from traditional medicinal plants [23], and CO [24] inhibit prostate cancer cell growth through AMPK activation, pointing to a relevant therapeutic role of AMPK in prostate cancer.

AMPK can be activated by canonical and non-canonical pathways [25]. The canonical pathway includes an increase in the AMP/ATP or ADP/ATP ratios and the phosphorylation of the catalytic α subunit by the liver kinase B1 (LKB1) [26], whereas the non-canonical mechanisms involve phosphorylation by other kinases, oxidative modification or the binding of long-chain fatty acyl-CoA esters [25].The phosphorylation of the AMPK α-subunit at Thr172 is tightly controlled by several kinases, including the fully validated AMPK upstream kinase LKB1; the calcium/calmodulin-dependent kinase kinase 2 (CaMKK2); and the less validated kinases TGFβ-activated kinase 1 (TAK1), mixed-lineage kinase 3 (MLK3) [27], calcium/calmodulin-dependent kinase kinase 1 (CaMKK1) and vaccinia virus-related kinase 1 (VRK1) [28], each one activated in different contexts that connect AMPK activation with the cell’s response. On the other hand, AMP binds to the regulatory γ subunit and allosterically enhances the phosphorylation of AMPK by LKB1 while inhibiting dephosphorylation by protein phosphatases [15].

In this study, we explored the involvement of LKB1 and CaMKK2 in the mechanism whereby capsaicin induces AMPK activation in prostate cancer cells. Our results showed that capsaicin could significantly inhibit proliferation and induce apoptosis in human prostate cancer cell lines that express LKB1 but not in the DU-145 cell line, which does not express LKB1. The investigation of the underlying mechanism reveals an involvement of the receptor TRPV1 in the activation of the LKB1/AMPK axis.

## 2. Materials and Methods

### 2.1. Materials

Capsaicin and STO-609 were purchased from Sigma-Aldrich (St. Louis, MO, USA). The psPAX2 vector was a gift from Didier Trono (Addgene, Watertown, MA, USA, plasmid #12260; http://n2t.net/addgene:12260, accessed on 24 January 2022; RRID:Addgene_12260), pCMV-VSV-G was a gift from Robert Weinberg [29] (Addgene plasmid #8454; http://n2t.net/addgene:8454, accessed on 24 January 2022; RRID:Addgene_8454), the pLKO.1-TRC cloning vector was a gift from David Root [30] (Addgene plasmid #10878; http://n2t.net/addgene:10878, accessed on 24 January 2022; RRID:Addgene_10878), pMDLg/pRRE was a gift from Didier Trono [31] (Addgene plasmid #12251; http://n2t.net/addgene:12251, accessed on 24 January 2022; RRID:Addgene_12251), pRSV-Rev was a gift from Didier Trono [31] (Addgene plasmid #12253; http://n2t.net/addgene:12253, accessed on 24 January 2022; RRID:Addgene_12253) and LentiV_Neo_LKB1 was a gift from Christopher Vakoc [32] (Addgene plasmid #108111; http://n2t.net/addgene:108111, accessed on 24 January 2022; RRID: Addgene_108111).

### 2.2. Cell Culture

PC3, DU-145 and LNCaP human prostate cancer cell lines were obtained from the American Type Culture Collection (ATCC CRL-1435, ATCC HTB-81 and ATCC CRL-1740, respectively) (American Type Culture Collection, Rockville, MD, USA). The cells were routinely grown in RPMI 1640 medium supplemented with 100 IU/mL penicillin G sodium, 100 µg/mL streptomycin sulfate, 0.25 µg/mL amphotericin B (Invitrogen, Paisley, UK) and 10% fetal bovine serum. All cell lines were incubated at 37 °C in 5% CO_2_ and routinely tested for *Mycoplasma* infection. For treatment experiments, the cells were plated and grown for 24 h, the medium was then replaced with serum-free RPMI 1640 and then incubated with different treatments for the indicated times. The cells were used at passages 4–20.

### 2.3. Cell Viability

Cell proliferation/viability was determined by an MTT assay (Bio-Rad, Richmond, CA, USA). The assay was performed in 12-well plates, according to the manufacturer’s instructions (5 × 10^3^/well). The absorbance was measured at 490 and 650 nm using an iMark™ Absorbance Reader from Bio-Rad (Richmond, CA, USA).

### 2.4. Flow Cytometry for Apoptosis

Apoptosis was evaluated at 24 h following treatment using an Annexin V-fluorescein isothiocyanate (FITC) apoptosis detection kit (BD Biosciences, San Diego, CA USA) according to the manufacturer’s instructions. Data acquisition and analysis were performed in a MACSQuant^®^ analyzer flow cytometry system (Miltenyi Biotec, Bergisch Gladbach, Germany) using the MACSQuantify software (Miltenyi Biotec, Bergisch Gladbach, Germany). A total of 10 × 10^3^ events were collected for each sample.

### 2.5. Western Blot

Proteins for Western blotting were isolated by lysing cells in lysis buffer (50 mM Tris pH 7.4, 0.8 M NaCl, 5 mM MgCl2, 0.1% Triton X-100) containing protease inhibitor and a phosphatase inhibitor cocktail (Roche Diagnostics, Mannheim, Germany), incubated on ice for 15 min and cleared by microcentrifugation. Twenty micrograms of total protein/lane were separated by SDS-polyacrylamide gel electrophoresis (SDS-PAGE) and then transferred onto a PVDF membrane. The membranes were incubated overnight at 4 °C with primary antibodies. After washing in T-TBS, the membranes were incubated with peroxidase-conjugated anti-mouse or anti-rabbit secondary antibodies (1:5000) for 2 h at room temperature. The immune complex was visualized with an ECL system (Cell Signaling Technology, Danvers, MA, USA). Protein expression levels were quantified using Image J (National Institutes of Health, Bethesda, MD USA) and were expressed as fold changes relative to the control treatment. The primary antibodies (anti-p-AMPKα1-thr172, p-ACC-ser79 and pLKB1-ser428) and the antibodies against the corresponding total forms were obtained from Cell Signaling Technology (Danvers, MA, USA). TRPV1 was obtained from Thermo Scientific (Waltham, MA, USA). Peroxidase-labeled secondary anti-mouse IgG was from Sigma-Aldrich (St. Louis, MO, USA) and anti-rabbit IgG was from Cell Signaling Technology (Danvers, MA, USA).

### 2.6. siRNA Transfections

Cells were transfected in 1 mL OptiMEM containing 4 μg Lipofectamine iMax (Invitrogen, Carlsbad, CA, USA) with 100 nM LKB1-specific small interfering RNA (siRNA) duplexes (5′-GUACUUCUGUCAGCUGAUUdTdT-3′ and 5′-AAUCAGCUGACAGAAGUACdTdT-3′) (Sigma-Aldrich, St. Louis, MO, USA) or scrambled RNA (control), according to the manufacturer’s protocols (Invitrogen, Carlsbad, CA, USA). At 72 h after transfection, the medium was removed and replaced with RPMI. At the indicated time points after transfection, the cells were used for MTT cell viability assays or Western blot analysis.

### 2.7. Lentivirus Transduction

The lentiviral transduction system was used to generate cell lines with TRPV1 silencing or LKB1 overexpression. Lentivirus was produced in HEK293T cells by transfecting the plasmids of interest with helper plasmids. To generate the viruses to silence TRPV1, the following mixture was added to a 10 cm dish of HEK293T cells at 70% confluence: 5 μg of psPAX2, 3 μg of pCMV-VSV-G, 10 μg of pLKO.1-TRC cloning vector or pLKO.1-TRC cloning vector with shTRPV1 (shTRPV1 sequence was designed from the clone ID: TRCN0000044190, Sigma, St. Louis, MO, USA) and polyethylenimine (PEI) 1 mg/mL at a 3:1 ratio with the total concentration of the DNA in the mixture. On the other hand, to generate the viruses to overexpress LKB1, the mixture was the following: 5 μg of pMDLg/pRRE, 3 μg of pCMV-VSV-G, 2.5 μg of pRSV-Rev, 10 μg of plasmid LentiV_Neo_LKB1 and PEI 1mg/mL in the same relationship discussed above. At 6 h after transfection, the medium was changed to fresh medium, and after 48 h and 72 h after transfection, the supernatant with the viruses was collected, filtered through a 0.45 µm pore-size filter and used to infect PC3 and DU-145 cells, with the addition of polybrene (1 µg/mL) (Sigma, St. Louis, MO, USA) to increase the efficiency of the infection. After infection, the cells were amplified to a larger culture surface and 24 h later they were selected with 3 µg/mL puromycin (STEMCELL Technologies, Vancouver, BC, Canada) in the case of TRPV1 silencing or with 900 µg/mL G418 (Sigma, St. Louis, MO, USA) in the case of LKB1 overexpression.

### 2.8. Statistical Analysis

The statistical analysis of the results was performed with GraphPad Prism 9 software (GraphPad, San Diego, CA, USA) using a two-way Analysis of Variance (ANOVA) and Tukey’s multiple comparisons test or Sidak’s multiple comparisons test. The results were reported as mean ± SD of at least three independent experiments, as indicated in the figure captions. Data were considered significant when *p* ≤ 0.05.

## 3. Results

### 3.1. Inhibition of Prostate Cell Proliferation by Capsaicin

**Figure 1:**
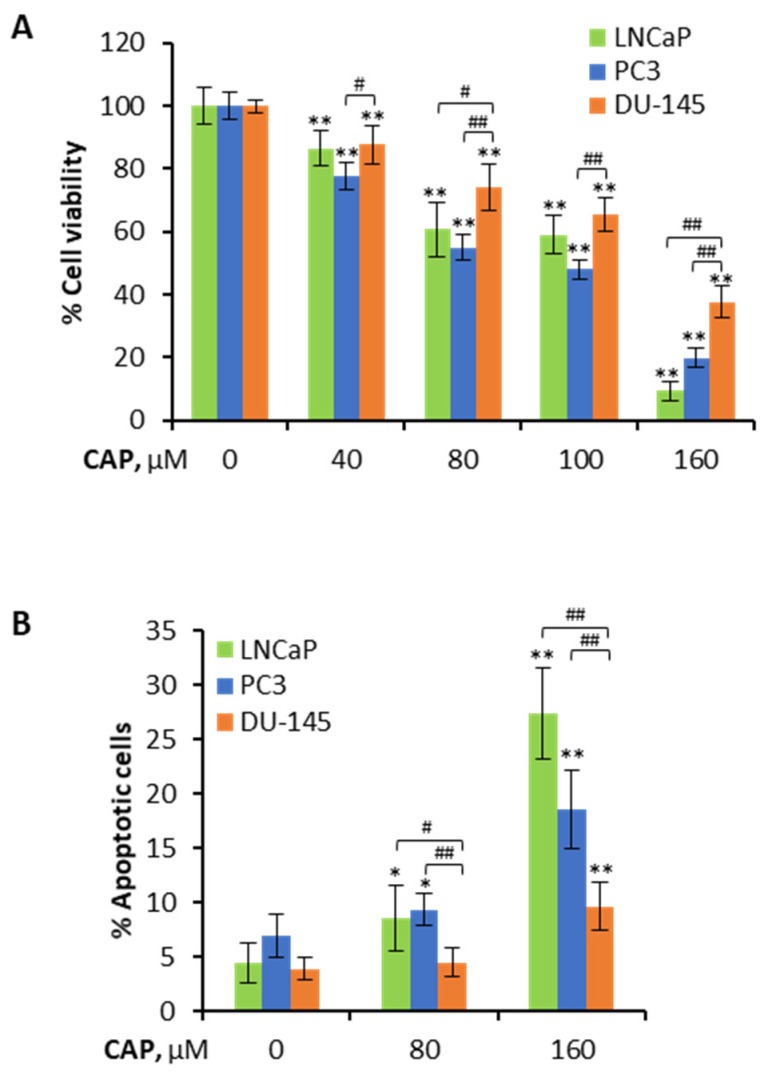
Lower antiproliferative effect of capsaicin on the prostate cancer DU-145 cell line compared to LNCaP and PC3 cell lines. (**A**) Effect of the different doses of capsaicin on prostate cancer PCa cell viability. Cells were treated with capsaicin at the indicated concentrations for 24 h. Cell viabilities were determined by MTT assay and are expressed as percentages of the control (DMSO treatment). (**B**) LNCaP, PC3 and DU-145 cells were treated with DMSO (control) or the indicated doses of capsaicin for 24 h and then stained with Annexin V and PI. The apoptotic cells (Annexin V-positive, PI-positive) are indicated as the percentage of gated cells. Bar graph represents the late apoptotic cells for each dose. Data are the mean ± SD. * *p* < 0.01 and ** *p* < 0.0001 indicate significant differences between the treated and control cells by two-way ANOVA and Tukey’s multiple comparisons test; # *p* < 0.001 and ## *p* < 0.0001 indicate significant differences between LNCaP and PC3 compared to DU-145. Experiments were run in triplicate and carried out at least five times on separate occasions.

We first investigated the antiproliferative effect of capsaicin on the prostate cancer cell lines LNCaP, PC3 and DU-145. As shown in Figure 1A, capsaicin reduced the cell viability of the three cell lines but was less potent in DU-145 cells, especially at the higher doses of 80 µM and 160 µM. The resistance of DU-145 cells to capsaicin was more clearly observed in apoptosis. While capsaicin induced apoptosis in 27% of LNCaP cells and 18% of PC3 cells, it only induced apoptosis in 9.6% of DU-145 cells (Figure 1B and Figure S1), pointing to a lower efficacy of capsaicin in DU-145 cells.

### 3.2. Capsaicin Activation of AMPK Depends on LKB1

**Figure 2:**
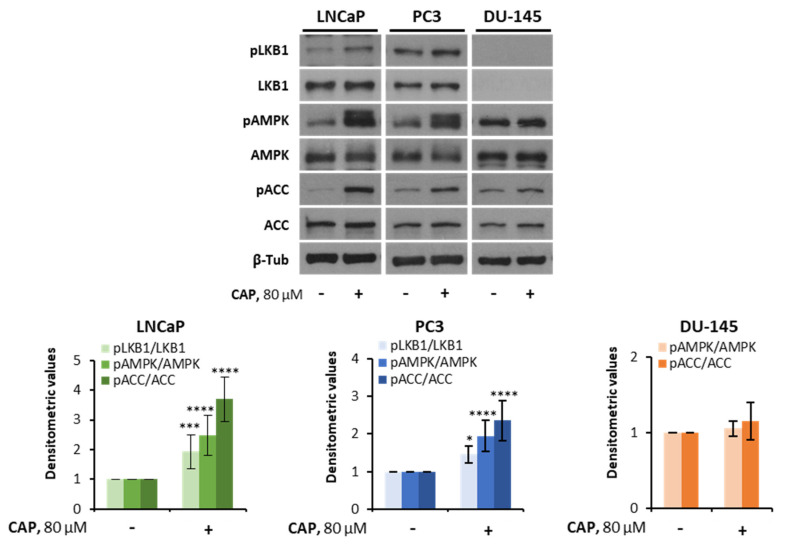
Capsaicin does not activate the LKB1/AMPK pathway in DU-145 cells. LNCaP, PC3 and DU-145 cells were treated with capsaicin for 1 h. The levels of the phosphorylated proteins and their total forms were determined by Western blot and β-tubulin (β-Tub) served as a loading control. A representative image of at least four experiments is shown. The densitometric analyses of the bands represent the mean ± SD. * *p* < 0.05, *** *p* < 0.001 and **** *p* < 0.001 indicate significant differences between the treated and control cells by two-way ANOVA and Tukey’s multiple comparisons test.

It has been reported that the prostate DU-145 cell line harbors a loss-of-function mutation in the STK11 gene encoding LKB1 [33]. Consequently, we questioned whether the anti-survival effect induced by capsaicin was dependent on LKB1. Therefore, we examined the ability of capsaicin to stimulate LKB1 in prostate cells. Treatment of LNCaP and PC3 cells with capsaicin increased LKB1 phosphorylation at Ser428, which is indicative of its activation (Figure 2). As previously mentioned, the DU-145 cells did not express LKB1; therefore, capsaicin did not induce its phosphorylation (Figure 2). It is worthy to note that capsaicin produced a notable increase in AMPK phosphorylation in the LKB1-expressing cell lines LNCaP and PC3, while it failed to activate AMPK in the LKB1-null DU-145 cell line (Figure 2). These results indicated that capsaicin activated AMPK in a LKB1-depenent fashion. To corroborate this notion, we examined the phosphorylation of the AMPK downstream target acetyl-CoA carboxylase (ACC), a key marker for determining AMPK activity in intact cells. As shown in Figure 2, ACC phosphorylation was increased in capsaicin-treated LNCaP and PC3 cells, but not in capsaicin-treated DU-145 cells. These findings support the notion that LKB1 plays a critical role in the mechanism whereby capsaicin activates AMPK in prostate cells.

**Figure 3:**
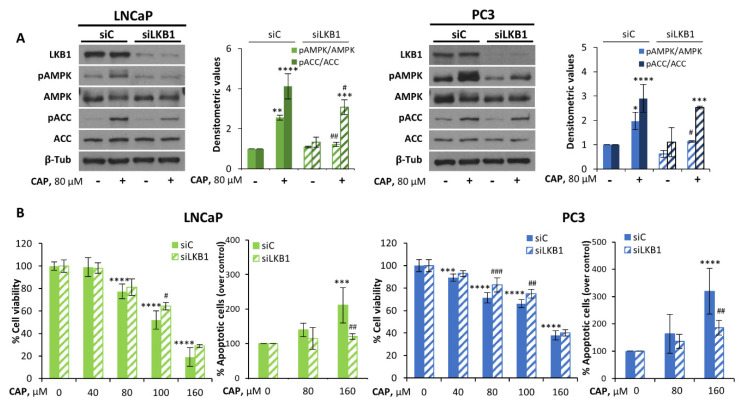
Capsaicin-induced activation of the AMPK pathway is dependent on LKB1 expression. LNCaP and PC3 cells were transfected with siControl (siC) or selective siLKB1 for 72 h. (**A**) LNCaP and PC3 cells were treated with 80 μM CAP for 1 h. The levels of the phosphorylated proteins and their total forms were determined by Western blot and β-Tub served as a loading control. A representative image of at least three experiments is shown. The densitometric analyses of the bands represent the mean ± SD. (**B**) Cell viabilities were determined by MTT assay and expressed as percentages of the controls (DMSO treatment). For apoptosis determination, LNCaP and PC3 cells were treated with DMSO (control) or the indicated doses of CAP for 24h and then stained with Annexin V and PI. Data are the mean ± SD of at least three different experiments. Data of the control of non-silenced and silenced cells were normalized to 100%. * *p* < 0.05, ** *p* < 0.01, *** *p* < 0.001 and **** *p* < 0.0001 indicate significant differences between treated cells and the control (DMSO treatment) by two-way ANOVA and Tukey’s multiple comparisons test; # *p* < 0.05, ## *p* < 0.01 and ### *p* < 0.001 indicate significant differences between non-silenced and silenced cells by two-way ANOVA and Tukey’s or Sidak’s multiple comparisons test.

To corroborate the involvement of LKB1 in capsaicin-induced AMPK activation, we knocked down LKB1 using siRNA in LNCaP and PC3 cells and assessed the ability of capsaicin to activate AMPK. As expected, the phosphorylation of AMPK was blocked in capsaicin-treated LKB1-knocked down LNCaP and PC3 cells (Figure 3A). Likewise, capsaicin-induced ACC phosphorylation decreased in LKB1 depleted cells; however, it was not completely abolished, suggesting that capsaicin might induce ACC phosphorylation by an alternative mechanism. Since we have previously demonstrated that capsaicin inhibits the proliferation of prostate cells through AMPK [2], and the activation of AMPK by capsaicin relies on LKB1, then LKB1 silencing should inhibit capsaicin’s antiproliferative effect. To confirm this notion, we analyzed the effect of capsaicin on LKB1-silenced cells. As shown in Figure 3B, LKB1 knockdown slightly but significantly reduced the antiproliferative effect of capsaicin in LNCaP and PC3 cells. Similar to DU145 cells, the preventive effect of LKB1 depletion was better observed in apoptosis (Figure 3B), suggesting that LKB1 plays a role in the apoptotic activity of capsaicin on prostate cells.

**Figure 4:**
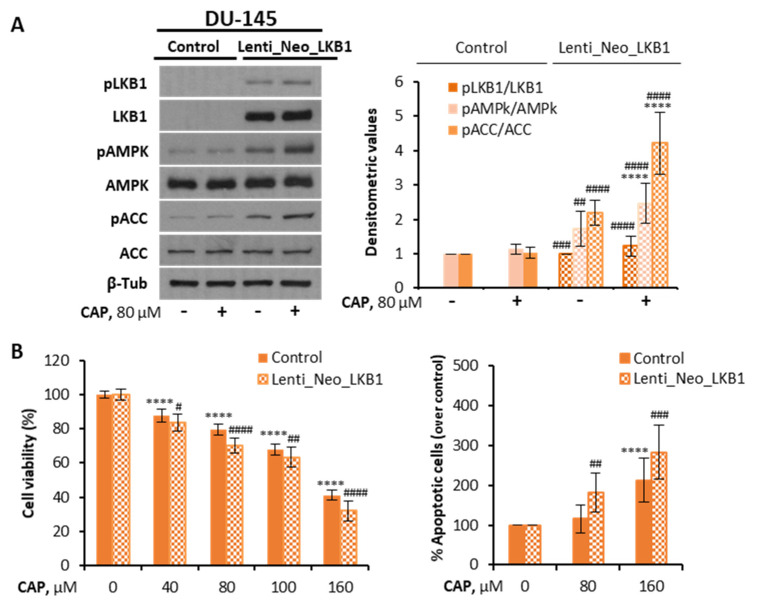
LKB1 expression in DU-145 cells promotes capsaicin-mediated activation of the LKB1/AMPK pathway. DU-145 cells were transfected with LKB1 by lentivirus-mediated transfection. (**A**) The cells were treated with CAP 80 μM for 1 h. The levels of the proteins were determined by Western blot and β-Tub served as a loading control. The densitometric analyses of the bands represent the mean ± SD of five different experiments. (**B**) Effect of LKB1 overexpression on cell viability and apoptosis. Cell viabilities were determined by MTT assay and expressed as percentages of the controls (DMSO treatment). For apoptosis determination, DU-145 cells were treated with DMSO (control) or the indicated doses of CAP for 24 h and then stained with Annexin V and PI. Data are the mean ± SD of eight different experiments. Data of the control of non-infected and infected cells were normalized to 100%. **** *p* < 0.0001 indicate significant differences between the treated cells and the control (DMSO treatment) by two-way ANOVA and Tukey’s multiple comparisons test; # *p* < 0.05, ## *p* < 0.01, ### *p* < 0.001 and #### *p* < 0.0001 indicate significant differences between non-infected and infected cells by two-way ANOVA and Tukey’s or Sidak’s multiple comparisons test.

As a further proof of the involvement of LKB1 in AMPK activation induced by capsaicin, we stably overexpressed LKB1 in DU-145 cells by lentiviral infection with particles carrying a Lenti_Neo_LKB1 vector. LKB1 overexpression was confirmed by Western blotting analyses (Figure 4A). Interestingly, when DU-145 cells expressed LKB1, capsaicin was able to activate AMPK (Figure 4A). The capsaicin treatment of DU-145 LKB1-expressing cells induced AMPK and ACC phosphorylation, both of which are phenomena indicative of AMPK activation (Figure 4A). We next investigated whether capsaicin inhibited cell proliferation following re-expression of LKB1 in LKB1-null tumor cell lines. The reintroduction of LKB1 into DU-145 cells slightly but significantly increased capsaicin-induced cell death and apoptosis (Figure 4B).

### 3.3. CaMKK2 Is Not Involved in AMPK Activation Induced by Capsaicin in Prostate Cells

**Figure 5:**
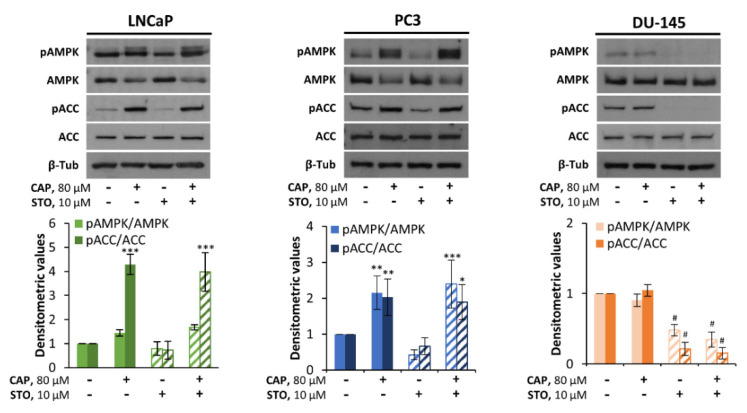
AMPK pathway activation by capsaicin is independent of CaMKK2. LNCaP, PC3 and DU-145 cells were pretreated with 10 µM STO-609 for half an hour and then incubated with 80 µM CAP for 1 h. The levels of proteins were determined by Western blot and β-tubulin served as a loading control. The densitometric analyses of the bands represent the mean ± SD of four different experiments. * *p* < 0.05, ** *p* < 0.01 and *** *p* < 0.001 indicate significant differences between the treated cells and the control (DMSO treatment) by two-way ANOVA and Tukey’s multiple comparisons test; # *p*<0.0001 indicates significant differences between the cells pretreated with STO-609 and the non-pretreated cells by two-way ANOVA and Tukey’s multiple comparisons test.

As stated in the introduction, AMPK can also be activated by the upstream kinase CaMKK2. To investigate the involvement of this kinase in the capsaicin-induced activation of AMPK, we used the selective and cell-permeable pharmacological CaMKK2 inhibitor STO-609. As shown in Figure 5, pre-treatment with STO-609 failed to prevent the phosphorylation of AMPK or ACC induced by capsaicin in LNCaP and PC3 cells. Intriguingly, STO-609 completely abolished the phosphorylation of either AMPK or ACC in the LKB1-null cell line DU-145 (Figure 5). These results indicate that CaMKK2 is not involved in the mechanism of action whereby capsaicin activates AMPK in prostate cells. However, CaMKK2 may be involved in the basal phosphorylation of AMPK in DU-145 cells, which do not express LKB1.

### 3.4. TRPV1 Is Required for LKB1 and AMPK Activation

**Figure 6:**
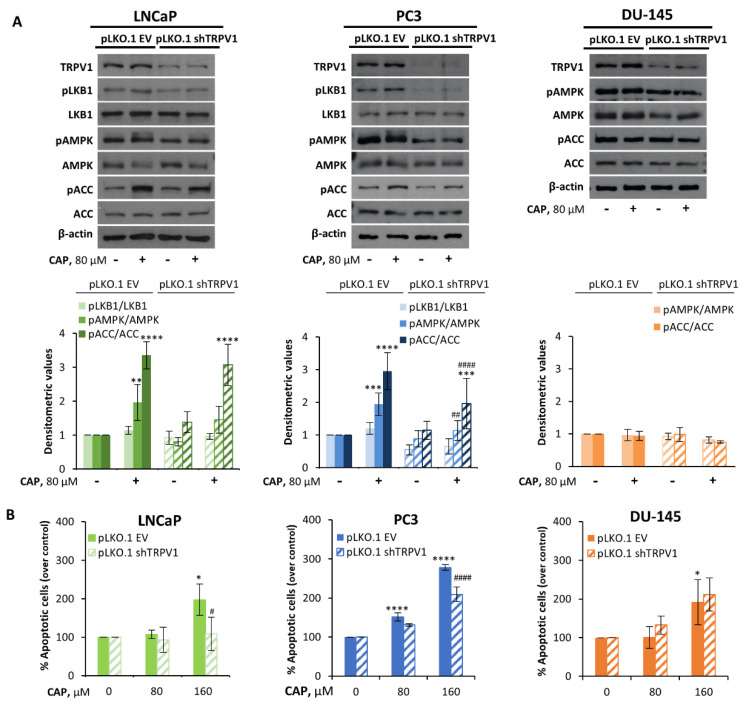
TRPV1 is required for LKB1 and AMPK activation. LNCaP, PC3 and DU-145 cells were transfected with shControl and shTRPV1 by lentivirus-mediated transfection. (**A**) LNCaP, PC3 and DU-145 cells were treated with CAP for 1h. The levels of the proteins were determined by Western blot and β-actin served as a loading control. The densitometric analyses of the bands represent the mean ± SD of five different experiments. (**B**) Effect of TRPV1 silencing on apoptosis. LNCaP, PC3 and DU-145 cells transfected with shControl or shTRPV1 were treated with DMSO (control) or the indicated doses of CAP for 24 h and then stained with Annexin V and PI. Data are the mean ± SD of at least two different experiments. Data of the control of non-silenced and silenced cells were normalized to 100% to appreciate the variations. * *p* < 0.05, ** *p* < 0.01, *** *p* < 0.001 and **** *p* < 0.0001 indicate significant differences between the treated cells and the control (DMSO treatment) by two-way ANOVA and Tukey’s multiple comparisons test; # *p* < 0.05, ## *p* < 0.01, #### *p* < 0.0001 indicate significant differences between non-silenced and silenced cells by two-way ANOVA and Tukey’s or Sidak’s multiple comparisons test.

Capsaicin effectively activates the transient receptor potential vanilloid 1 (TRPV1), a cation channel expressed in sensitive neurons as well as in other tissues, including the prostate and prostate cancer cells [34,35]. It has been recently discovered that TRPV1 plays essential roles in cancer tumorigenesis and development [36]. However, TRPV1 agonists may exert antitumor effects via a receptor-dependent or independent mechanism [36]. To examine the involvement of the TRPV1 channel in the mechanism of capsaicin-induced antitumor effect and AMPK activation, we knocked down TRPV1 expression by infection with lentiviral viruses carrying small hairpin RNA (shRNA). The most efficient sequence against TRPV1 was cloned into the lentiviral vector pLKO.1 and the resulting plasmid was used to produce viruses in HEK293T cells. Empty vector viruses (pLKO.1 EV) were used to infect control cells. The knockdown efficiency of TRPV1-specific shRNA was confirmed at the protein level by Western blotting analyses through comparison with those of a negative control (Figure 6A). As shown in Figure 6A, the genetic downregulation of TRPV1 decreased the phosphorylation level of LKB1 in LNCaP and PC3 cells, pointing to a connection between TRPV1 and LKB1 in prostate cells. Likewise, the increase in AMPK phosphorylation induced by capsaicin was also inhibited in TRPV1-downregulated cells (Figure 6A). However, in DU-145 cells, the genetic depletion of TRPV1 did not modify the phosphorylation of AMPK in the presence of capsaicin (Figure 6A), since these cells do not express LKB1. This shows that TRPV1 is required for LKB1 activation and that LKB1 is required for AMPK activation, highlighting a TRPV1/LKB1/AMPK signaling pathway in prostate cancer cells. The genetic depletion of TRPV1 had an impact on the induction of apoptosis by capsaicin, since in LNCaP- and PC3-TRPV1-shRNA-infected cells, a significant prevention in capsaicin-induced apoptosis was observed (Figure 6B). Nevertheless, in DU-145 cells, TRPV1 downregulation did not have any impact on apoptosis, as evidenced by the lack of effectiveness of capsaicin on this cell line (Figure 6B). These results indicate that the anti-proliferative effect induced by capsaicin in prostate cells is mediated by a TRPV1-LKB1-AMPK-dependent mechanism.

## 4. Discussion

Here, we demonstrated that capsaicin exerts antiproliferative effects in prostate cancer cells expressing LKB1 by a TRPV1 receptor-dependent mechanism. Capsaicin reduces cell viability and promotes apoptosis by the activation of the TRPV1/LKB1/AMPK axis. It was recently revealed that TRPV1 is involved in cancer development and progression, although the precise role that TRPV1 plays remains to be elucidated. Despite that fact that both the expression and activity of TRPV1 are altered in many tumors, there is still great confusion about its role in regulating cell fate. In fact, both agonists and antagonists may reveal anti-cancer effects, and the effect may function via or be independent of TRPV1 [36]. For instance, Amantini et al. demonstrated that treatment of urothelial cancer cells with capsaicin arrests cell cycle progression and triggers apoptosis in a TRPV1-dependent fashion [37]. In human renal carcinoma, capsaicin induces apoptosis that is reversed by the TRPV1 antagonist capsazepine, implying a receptor-mediated mechanism [38]. However, in hepatocarcinoma cells, Zhang et al. demonstrated that capsaicin enhances the antitumor activity of sorafenib independently of TRPV1 [39]. We have previously demonstrated that capsaicin exerts a synergistic antitumor effect with sorafenib in hepatocellular carcinoma cells and with docetaxel in prostate cancer cells through AMPK activation [2,40]. Our results from this study show that capsaicin activates AMPK in prostate cancer cells via a TRPV1/LKB1-dependent phosphorylation at Thr172.

Due to the important role of AMPK in regulating cell fate and metabolism, the phosphorylation of AMPK at Thr172 is tightly regulated by upstream kinases and phosphatases. However, the predominant upstream AMPK kinase in the prostate has not been well established [41]. Although the role of CaMKK2 in prostate cancer has been recently revealed, it does not appear to have an essential effect on growth regulation [42]. CaMKK2 levels have been found to be elevated in clinical samples of prostate cancer, where it regulates cancer cell growth [43]. In addition, androgens regulate the expression of CaMKK2 in prostate cells harboring the CaMKK2 promoter, an androgen-responsive element [42,44]. However, protein synthesis is unperturbed by targeting the AR-CaMKK2-AMPK pathway in prostate cancer cells, suggesting that although CaMKK2 stimulates glycolysis, it has no significant effects on biosynthesis [42]. This agrees with our results showing that AMPK activation by capsaicin is independent of CaMKK2.

On the contrary, our results show that capsaicin activates AMPK in prostate cells by a LKB1-dependent pathway that regulates cell growth. Shackelford et al. demonstrated several years ago that the drug phenformin, a biguanide chemically related to metformin, was more effective in the treatment of non-small cell lung cancer (NSCLC) if the tumors lacked a functional LKB1-AMPK pathway, suggesting an LKB1-AMPK-independent mechanism of action [45]. However, this was not the case in our work, since capsaicin inhibited cell growth more efficiently in PC3 and LNCaP cells than in DU145 cells, which lack LKB1. Moreover, the rescue of LKB1 expression by lentiviral infection in DU-145 cells allowed AMPK activation by capsaicin. The activation of AMPK by LKB1 in prostate LNCaP and PC3 cells has been also observed by Yan et al. [24], who reported that the treatment of prostate cells with CO provoked an increase in LKB1 expression and AMPK activation, and significantly suppressed tumor growth.

**Figure 7:**
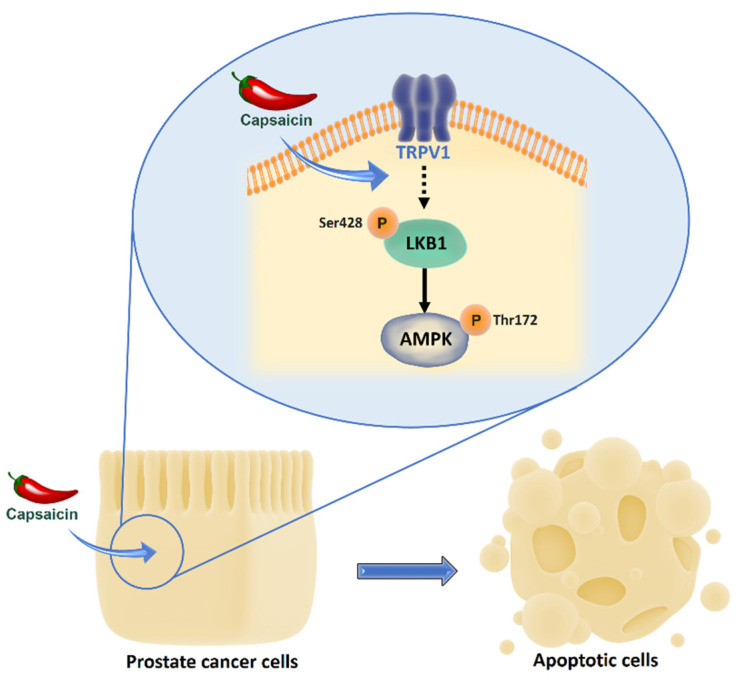
Capsaicin promotes apoptosis in prostate cells by activating the TRPV1/LKB1/AMPK axis.

Our work describes a novel connection between the capsaicin receptor TRPV1 and the LKB1/AMPK pathway in prostate cells (Figure 7). Our novel finding that TRPV1 acts as upstream regulator of LKB1 uncovers a molecular pathway linking the cation channel with cell fate. In agreement with our results, Li et al. recently proposed that TRPV channels may activate AMPK independently of AMP, and that the genetic depletion of TRPV1 blocks AMPK activation, which is indicative of the physical requirement of TRPV1 to activate AMPK [46]. The TRPV1 channel induces lysosomal AMPK activation in low glucose conditions through the formation of an AXIN-based super-complex on the lysosomal surface that allows LKB1 to phosphorylate and activate AMPK. According to Maiese K. [47], TRPV1 receptors do not rely entirely upon calcium signaling to affect cellular biology, but also have a close relationship with AMPK, mTOR and protein kinase B (Akt), which agrees with our results. Here, we show that TRPV1 is connected with AMPK via LKB1, although we do not know the mechanism whereby TRPV1 is linked to LKB1 in prostate cells. Further research to unravel the underlying pathway will shed light on the role of TRPV1 in growth regulation.

Altogether, our results indicate that the activation of the TRPV1/LKB1/AMPK pathway by capsaicin results in a significant decrease in cell proliferation, suggesting that TRPV1-targeted pharmaceutical interventions may be exploited to suppress the growth of prostate tumors.

## Data Availability

The data used to support the findings of this study are deposited in https://data.mendeley.com/datasets/w77g7vdsjp/draft?a=92c84270-6569-4d39-bed4-9278ca2257fe, accessed on 24 January 2022.

## Supplementary Materials

The following supporting information can be downloaded at: https://www.mdpi.com/article/10.3390/pharmaceutics14020329/s1, Figure S1: Capsaicin induces apoptosis in LNCaP and PC3 cells but not in DU-145 cells.
