# Supplementary material for: The Natural Chemotherapeutic Capsaicin Activates AMPK through LKB1 Kinase and TRPV1 Receptors in Prostate Cancer Cells
Source: Pharmaceutics. 2022 Jan 29;14(2):329. doi: 10.3390/pharmaceutics14020329 (PMC8880011; doi:10.3390/pharmaceutics14020329)
Supplement: Supplementary file 1 [file pharmaceutics-14-00329-s001.zip › pharmaceutics-1547322-SI.pdf]

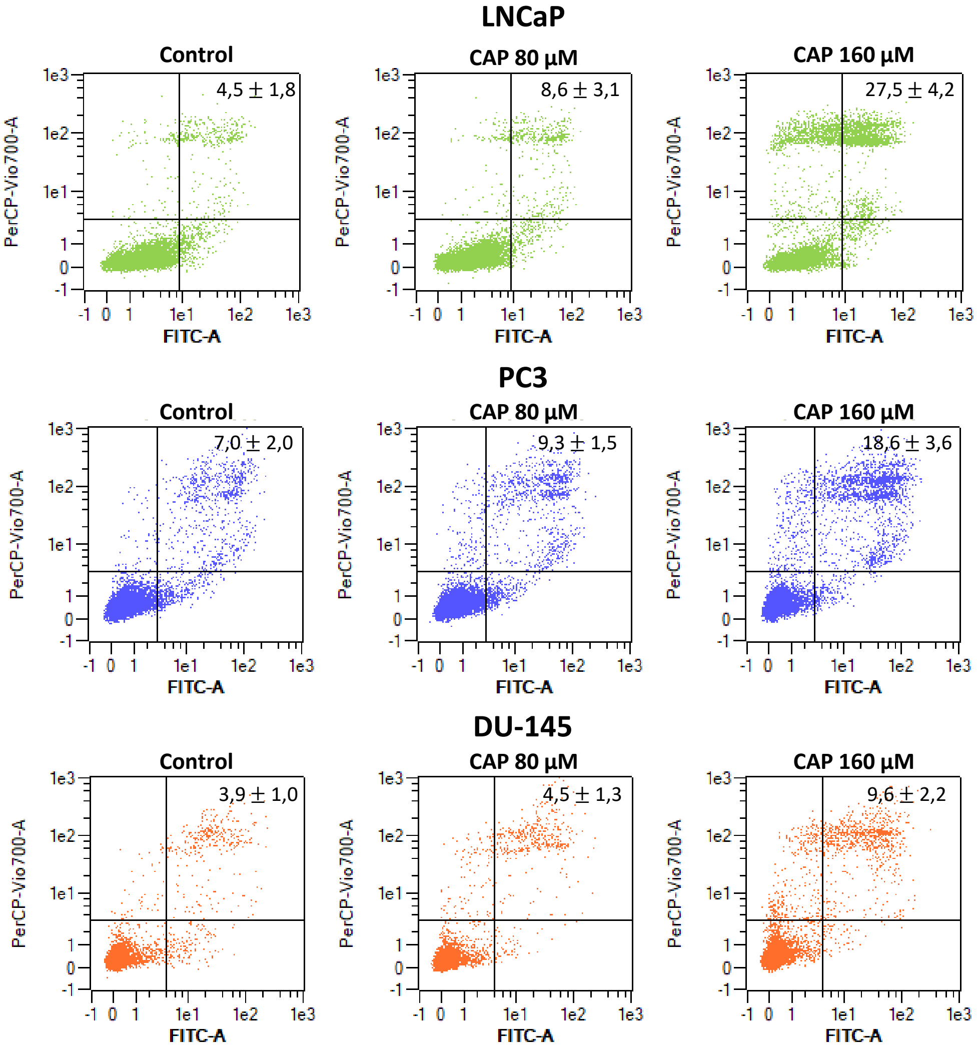


**Figure S1. Capsaicin induces apoptosis in LNCaP and PC3 cells but not in DU-145 cells.** LNCaP, PC3 and DU-145 cells were treated with DMSO (control) or the indicated doses of capsaicin for 24h and then stained with Annexin V and PI. The graphs represent PI fluorescence (Y axe) versus Annexin V fluorescence (X axe).
